# Bacterial Thymidine Kinase as a Non-Invasive Imaging Reporter for *Mycobacterium tuberculosis* in Live Animals

**DOI:** 10.1371/journal.pone.0006297

**Published:** 2009-07-16

**Authors:** Stephanie L. Davis, Nicholas A. Be, Gyanu Lamichhane, Sridhar Nimmagadda, Martin G. Pomper, William R. Bishai, Sanjay K. Jain

**Affiliations:** 1 Center for Tuberculosis Research, Johns Hopkins University School of Medicine, Baltimore, Maryland, United States of America; 2 Department of Pediatrics, Johns Hopkins University School of Medicine, Baltimore, Maryland, United States of America; 3 Department of Medicine, Johns Hopkins University School of Medicine, Baltimore, Maryland, United States of America; 4 Department of Radiology, Johns Hopkins University School of Medicine, Baltimore, Maryland, United States of America; University of Minnesota, United States of America

## Abstract

**Background:**

Bacteria can be selectively imaged in experimentally-infected animals using exogenously administered 1-(2′deoxy-2′-fluoro-β-D-arabinofuranosyl)-5-[^125^I]-iodouracil ([^125^I]-FIAU), a nucleoside analog substrate for bacterial thymidine kinase (TK). Our goal was to use this reporter and develop non-invasive methods to detect and localize *Mycobacterium tuberculosis*.

**Methodology/Principal Findings:**

** We engineered a *M. tuberculosis* strain with chromosomally integrated bacterial TK under the control of *hsp60* - a strong constitutive mycobacterial promoter. [^125^I]FIAU uptake, antimicrobial susceptibilities and *in vivo* growth characteristics were evaluated for this strain. Using single photon emission computed tomography (SPECT), *M. tuberculosis* P_hsp60_ TK strain was evaluated in experimentally-infected BALB/c and C3HeB/FeJ mice using the thigh inoculation or low-dose aerosol infection models. *M. tuberculosis* P_hsp60_ TK strain actively accumulated [^125^I]FIAU *in vitro*. Growth characteristics of the TK strain and susceptibility to common anti-tuberculous drugs were similar to the wild-type parent strain. *M. tuberculosis* P_hsp60_ TK strain was stable *in vivo* and SPECT imaging could detect and localize this strain in both animal models tested.

**Conclusion:**

We have developed a novel tool for non-invasive assessment of *M. tuberculosis* in live experimentally-infected animals. This tool will allow real-time pathogenesis studies in animal models of TB and has the potential to simplify preclinical studies and accelerate TB research.

## Introduction

Current tools for monitoring tuberculosis (TB) in preclinical animal studies are costly and time consuming. Significant resources are required for processing (animal sacrifice, organ harvesting) hundreds of animals per study. Since *Mycobacterium tuberculosis* grows slowly, colony forming unit (CFU) counts and subsequent results are not available for at least 4-weeks after the completion of the study. Moreover, since the entire organ is generally homogenized and different animals are sacrificed at each time-point; lesion-specific responses in the same animal can also never be assessed.

While mouse chemotherapy is generally concordant with human studies, and animal-to-animal variability is low owing to the availability of inbred strains, a significant drawback of the standard mouse model is the lack of caseation – the hallmark of human disease. As new drugs and vaccines are developed which target persistence, characterization of alternative models that develop microenvironments which are absent in mouse lungs, but which may be relevant to human TB lesions, is fundamental to preclinical assessment. Therefore, it may be essential to perform cross-species studies in larger animal models such as guinea pigs, rabbits, non-human primates (NHP) which develop microenvironments relevant to human TB [1]. Animal-to-animal variability is a more serious concern with these larger, more expensive species, which are generally not available as inbred strains. The need for non-invasive biomarkers that can monitor response in the same group of animals and therefore significantly reduce the numbers of animals required per study will be essential to cost-effectively conduct these studies in the larger and more expensive animals.

Bacteria can be selectively imaged in experimentally-infected mice using exogenously administered 1-(2′deoxy-2′-fluoro-β-D-arabinofuranosyl)-5-[^125^I]-iodouracil ([^125^I]FIAU), a nucleoside analog substrate for bacterial TK [2]. TK phosphorylates FIAU, leading to accumulation within the bacteria. However, since FIAU is a poor substrate for mammalian TK, the radiotracer selectively labels bacteria. These bacteria can therefore be imaged *in situ* after an intravenous administration of [^125^I]FIAU using single photon emission computed tomography (SPECT) in conjunction with computed tomography (CT). Since radio-pharmaceutical based imaging provides a comprehensive 3-dimensional (3-D) assessment of the whole organ, it also generally correlates closely with the overall disease. In this study, we adapted the thymidine kinase reporter system for *M. tuberculosis*. Since mycobacteria lack TK, we engineered and characterized a *M. tuberculosis* strain expressing TK and used it to detect and localize *M. tuberculosis* in live animals *in situ*.

## Methods

### *M. tuberculosis* P_hsp60_ TK strain

Using the Invitrogen Gateway Cloning system we constructed a vector, pGS400H, for efficient delivery of a single copy of DNA to the *attB* site in the mycobacterial genome. Bacterial TK from *Escherichia coli* (Kind gift of Kwang Sik Kim, Johns Hopkins University) was PCR amplified and cloned into pGS400H downstream of *P_hsp60_* - a highly active constitutive mycobacterial promoter [3]. *M. tuberculosis* H37Rv strain was transformed with this vector and colonies selected on hygromycin plates using standard methods. Cloning was confirmed using PCR and Southern blotting.

### *M. tuberculosis* strains and media

*M. tuberculosis* H37Rv and *M. tuberculosis* P_hsp60_ TK strains were grown to mid-log phase in plastic roller bottles or as shaken cultures in plastic tubes at 37°C in Middlebrook 7H9 liquid broth (Difco Laboratories) supplemented with oleic acid albumin dextrose catalase (OADC) (Becton Dickinson), 0.5% glycerol, and 0.05% Tween 80. Hygromycin (50 µg/ml) was used for all *M. tuberculosis* P_hsp60_ TK broth cultures. Before thigh injections, mycobacteria were washed and resuspended in phosphate buffered saline (PBS), and their optical densities at 600 nm (OD_600_) were adjusted to achieve the required bacterial density. In addition, 100 µL from each inocula were plated to determine the colony forming units (CFU) counts. *M. tuberculosis* were plated onto Middlebrook 7H11 selective plates (Becton Dickinson).

### *In vitro* [^125^I]FIAU uptake

Equal numbers of *M. tuberculosis* H37Rv (wild-type) or *M. tuberculosis* P_hsp60_ TK strains were incubated with 1 µCi/ml of [^125^I]FIAU at 37°C in Middlebrook 7H9 liquid broth for 6 and 24 hours. At each specified time-point equal aliquots were withdrawn from the cultures and washed 3 times to remove free [^125^I]FIAU in the media. Each pellet was resuspended in PBS in 1.5 ml Eppendorf tubes and disinfected with Lysol overnight. The activity for each Eppendorf was measured using an automated gamma counter (1282 Compugamma CS Universal gamma counter, LKB Wallac). Each assay was performed in triplicate.

### Antimicrobial susceptibilities

*M. tuberculosis* H37Rv and *M. tuberculosis* P_hsp60_ TK strains were tested against common anti-TB drugs (streptomycin, ethambutol, INH, rifampin and moxifloxacin) and FIAU (non-radioactive) using the standard broth dilution method. Briefly, mid-log phase bacteria were diluted to a concentration of 100,000 organisms/ml and grown at 37°C in Middlebrook 7H9 liquid broth (without Tween 80) containing different concentrations of antibiotics. Mean inhibitory concentrations (MIC) were determined 10–14 days later. Each assay was performed in triplicate.

### Animal infection

5–6 week old female BALB/c (Charles River) or C3HeB/FeJ (Jackson Laboratory) mice were used. Equal numbers of *M. tuberculosis* H37Rv or *M. tuberculosis* P_hsp60_ TK strains in 50–100 µl of PBS were inoculated into either thigh of BALB/c mice.

C3HeB/FeJ mice were low-dose aerosol infected with equal numbers of either *M. tuberculosis* H37Rv or *M. tuberculosis* P_hsp60_ TK strains using the Inhalation Exposure System (Glas-Col). Three mice were sacrificed at 1-day and 2-weekly intervals thereafter, and the lungs homogenized and plated for CFU to determine the number of bacilli implanted and bacterial growth respectively. Using TK specific primers and colony PCR, the TK gene was amplified from randomly picked *M. tuberculosis* P_hsp60_ TK colonies. Colonies obtained from *M. tuberculosis* wild-type infected mice and pGS400H vector were used as negative and positive controls respectively.

### Bio-containment and anesthesia

All live *M. tuberculosis* infected animals were imaged within a sealed bio-containment device developed by our laboratory. An unbreakable and transparent centrifuge bottle with a gasket screw cap (Nalgene) and holes for passage of gases was used for this purpose. Two 0.22 µm filters were used both at the inlet and the outlet to contain the bacteria within the device. A standard small animal anesthesia machine was used to deliver an Isoflurane (Henry Schein) oxygen mixture during transport and imaging. Animals were anesthetized and sealed inside the bio-containment device in the BSL-3 facility. The external surface of the bio-containment device was decontaminated and transported to the imaging suite. During prolonged anesthesia (>20 min), an infrared thermometer and a heat-lamp were used to measure and maintain ambient air temperature inside the bio-containment device.

### Imaging

BALB/c mice were imaged 3 and 12 hours after the thigh inoculation while C3HeB/FeJ mice were imaged at 6 or 8 weeks after the low-dose aerosol infection. Before imaging, each mouse was weighed, injected with 1 mCi of [^125^I]FIAU via the tail-vein and enclosed in the bio-containment device. Mice were imaged 3–24 hours post-injection using the X-SPECT (Gamma Medica) or NanoSPECT/CT (BIOSCAN) Small Animal SPECT/CT imagers with 45 minutes static acquisitions with 128 projections, or 60 minutes with 48 projections respectively. A CT scan was also performed at the same time. All images were scaled to the same level for comparison. SPECT images were reconstructed and co-registered with CT images using Amira (Visage Imaging). After imaging, lung tissues from *M. tuberculosis* H37Rv (wild-type) or *M. tuberculosis* P_hsp60_ TK infected animals were also disinfected and analyzed using the automated gamma counter.

All protocols were approved by the Animal Care and Use Committee, Bio-safety and Radiation-safety offices at Johns Hopkins University.

#### Statistical analysis

Statistical comparison between groups were performed using one tail distribution, two sample, unequal variance t-test in Excel 2007 (Microsoft). Data are presented as mean±standard deviation throughout the study.

## Results

### *M. tuberculosis* P_hsp60_ TK strain actively accumulates [^125^I]FIAU

**Figure 1:**
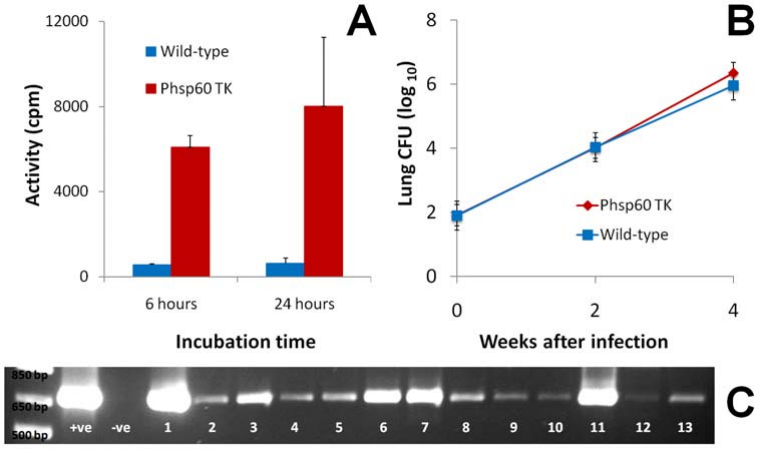
Characteristics of the *Mycobacterium tuberculosis* P_hsp60_ TK strain. A. Equal numbers of *M. tuberculosis* H37Rv (wild-type) or *M. tuberculosis* P_hsp60_ TK strains were incubated with 1 µCi/ml of [^125^I]FIAU at 37°C in Middlebrook 7H9 liquid broth for 6 and 24 hours. At each specified time-point equal aliquots were withdrawn from the cultures and washed 3 times to remove the media. Each pellet was resuspended in PBS in 1.5 ml Eppendorf tubes and disinfected with Lysol overnight. The activity for each Eppendorf was measured using a gamma counter. *M. tuberculosis* P_hsp60_ TK strain actively accumulates [^125^I]FIAU *in vitro*. Mean uptake activity is 6086 (±536) and 8011 (±3233) counts per minute (cpm) at 6 and 24 hours for the *M. tuberculosis* P_hsp60_ TK strain which is significantly more than 549 (±50) and 615 (±260) cpm for the wild-type parent strain (p<0.03). B. We evaluated the *in vivo* growth characteristics of *M. tuberculosis* P_hsp60_ TK strain and compared it to the wild-type parent strain. Both strains grow similarly in the lungs of C3HeB/FeJ mice after a low-dose aerosol infection. C. Using TK specific primers and colony PCR, the TK gene is reliably amplified from all the randomly picked *M. tuberculosis* P_hsp60_ TK colonies (n = 13) obtained from lung homogenates 8-weeks after an aerosol infection (panel B). +ve and –ve refer to the positive (pGS400H) and negative (*M. tuberculosis* wild-type) controls respectively.

We were successful in engineering a strain of *M. tuberculosis* H37Rv expressing TK under the control of *P_hsp60_* - a highly active constitutive mycobacterial promoter. As shown in Figure 1A, *M*. *tuberculosis* P_hsp60_ TK strain actively accumulated [^125^I]FIAU *in vitro*. Mean uptake activity was 6086±536 and 8011±3233 counts per minute (cpm) at 6 and 24 hours for the *M. tuberculosis* P_hsp60_ TK strain which were significantly more than 549±50 and 615±260 cpm for the *M. tuberculosis* wild-type parent strain (p<0.03).

### Antimicrobial susceptibilities for *M. tuberculosis* P_hsp60_ TK strain are similar to the wild-type strain

Since we plan to develop the *M. tuberculosis* P_hsp60_ TK strain as a reporter for bacterial burden *in situ* including TB drug development, we wanted to evaluate whether this strain has any alteration in antimicrobial susceptibility to common anti-TB drugs. Table 1 shows the MICs for 5 common anti-TB drugs for *M. tuberculosis* P_hsp60_ TK strain and the *M. tuberculosis* wild-type strain. As shown, there is no significant difference between the susceptibility patterns for either strain. Since FIAU may inhibit bacterial growth of strains expressing TK [2], we also wanted to evaluate whether *M. tuberculosis* P_hsp60_ TK strain would be inhibited by non-radioactive FIAU at concentrations achieved *in vivo*. However, FIAU at up to 8 µg/ml (hundreds of fold higher than achieved *in vivo* after a standard animal dose), did not inhibit the growth of *M. tuberculosis* P_hsp60_ TK strain.

**Table 1:** Antimicrobial susceptibilities for *Mycobacterium tuberculosis* P_hsp60_ TK strain are similar to the wild-type parent strain.

Antibiotic	*M. tuberculosis*_Phsp60_ TK strain	*M. tuberculosis* wild-type strain
Streptomycin	1	1
Ethambutol	1	2
INH	0.06	0.06
Rifampin	0.25	0.5
Moxifloxacin	0.5	0.5

*M. tuberculosis* H37Rv and *M. tuberculosis* P_hsp60_ TK strains were tested against common anti-TB drugs (streptomycin, ethambutol, INH, rifampin and moxifloxacin) using the standard broth dilution method. Mid-log phase bacteria were diluted to a concentration of 100,000 organisms/ml and grown at 37°C in Middlebrook 7H9 liquid broth (without Tween 80) containing different concentrations of antibiotics. Mean inhibitory concentrations (MIC) were determined 10–14 days later. As shown, there is no significant difference between MICs for 5 common anti-TB drugs for either strain.

### *M. tuberculosis* P_hsp60_ TK strain is stable *in vivo* and its growth characteristics are similar to the wild-type strain

*In vitro* growth of the *M. tuberculosis* P_hsp60_ TK strain was similar to the wild-type parent strain. Next we evaluated whether the *in vivo* growth characteristics of *M. tuberculosis* P_hsp60_ TK strain were also similar to the wild-type parent strain. Figure 1B shows that both strains grow similarly in the lungs of C3HeB/FeJ mice after a low-dose aerosol infection. Using TK specific primers and colony PCR, the TK gene was reliably amplified from all the randomly picked *M. tuberculosis* P_hsp60_ TK colonies (n = 13) obtained from lung homogenates 8-weeks after aerosol infection (Figure 1C) but not from the negative control.

### [^125^I]FIAU-SPECT can detect and localize *M. tuberculosis* P_hsp60_ TK strain *in situ*

**Figure 2:**
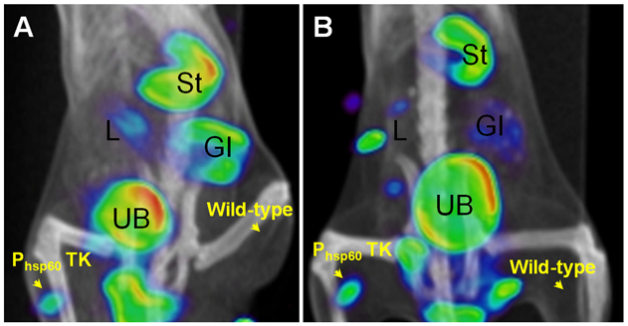
[^125^I]FIAU-SPECT can detect and localize *Mycobacterium tuberculosis* P_hsp60_ TK strain *in situ*. *M. tuberculosis* H37Rv wild-type or P_hsp60_TK strain (∼10^8^ CFU) were inoculated into either thighs of a BALB/c mouse. 1 mCi of [^125^I]FIAU was injected via a tail vein injection. Panels A and B show the fused SPECT and CT images 3 and 12 hours after the [^125^I]FIAU injection. Signal is detected at both time-points in the thigh inoculated with *M. tuberculosis* P_hsp60_TK strain but not in the thigh inoculated with the wild-type strain. As expected, SPECT signal is detected in several tissues [liver/gall bladder (L), stomach (St), gastrointestinal tract (GI), urinary bladder (UB)] that either metabolize or excrete FIAU or its iodinated derivatives.

*M. tuberculosis* wild-type or *M. tuberculosis* P_hsp60_TK strain **(∼**10^8^ CFU) were inoculated into either thigh of a BALB/c mouse. Before imaging, 1 mCi of [^125^I]FIAU was injected into each mouse via a tail vein injection. As shown in Figure 2, SPECT signal was detected as early as 3 hours from the thigh inoculated with *M. tuberculosis* P_hsp60_TK strain but not from the thigh with the wild-type strain. As expected, SPECT signal was detected in several tissues (liver, gall bladder, stomach, gastrointestinal tract, urinary bladder) that either metabolize or excrete FIAU or its iodinated derivatives [2], [4].

**Figure 3:**
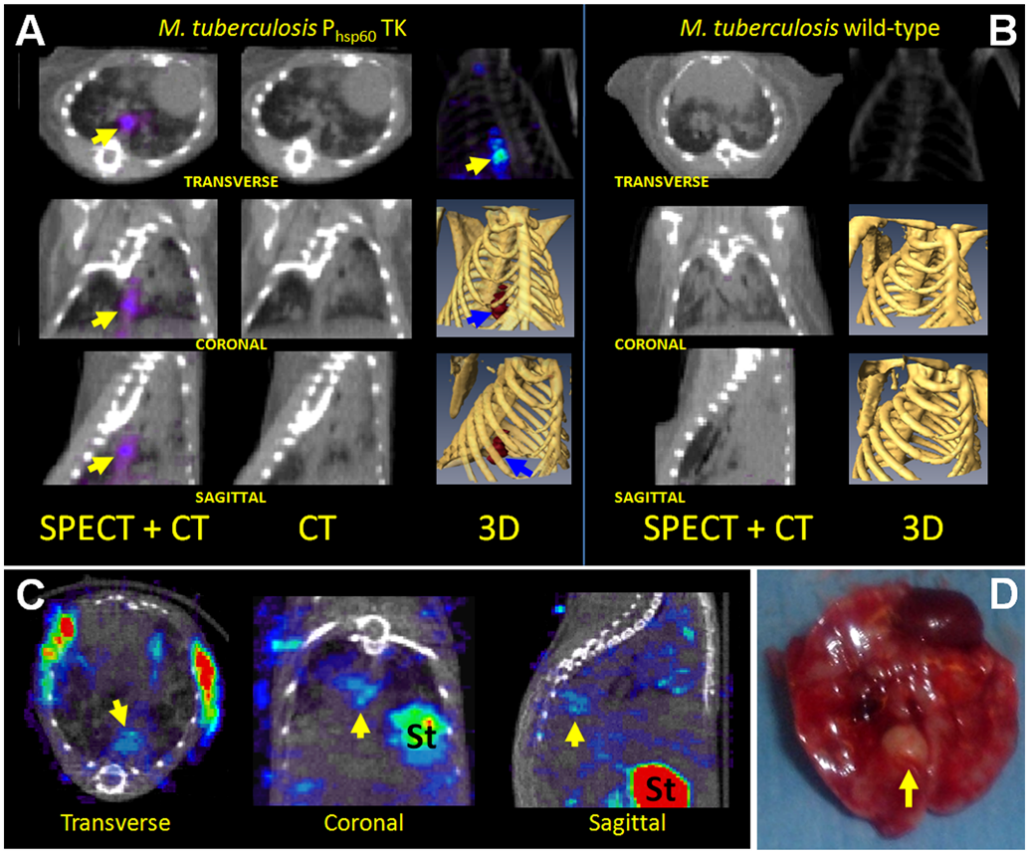
*Mycobacterium tuberculosis* P_hsp60_ TK signal localizes to caseating granulomas in lungs of C3HeB/FeJ mice. A. [^125^I]FIAU-SPECT/CT images from *M. tuberculosis* P_hsp60_TK infected C3Heb/FeJ mice 6-weeks after a low-dose aerosol infection. 3-D co-registered SPECT (blue/red) and CT (yellow) images are also shown. SPECT signal co-localizes to TB granulomas in the lungs of *M. tuberculosis* P_hsp60_TK infected mouse (arrows). B. Though extensive lung disease is present, images from *M. tuberculosis* wild-type infected mouse show no SPECT activity in the lungs. C. *M. tuberculosis P_hsp60_*TK SPECT signal localizes to a TB granuloma (arrows) in C3Heb/FeJ mice imaged 8-weeks after infection. D. The same lung and TB granuloma (arrow) is shown post-mortem. *Ex-vivo* radioactivity in the selected granuloma was 5318 counts per minute per milligram of tissue compared with 2364 in lung tissue outside of the granuloma and 856 in lung tissue from the animal infected with *M. tuberculosis* wild-type strain (St = Stomach).

Next we evaluated whether the *M. tuberculosis* P_hsp60_ TK strain could be visualized in lungs of mice that develop caseating lesions. Day 1 lung implantations after a low-dose aerosol infection were 1.36±0.17 and 1.95±0.07 log_10_ CFU for mice infected with the *M. tuberculosis* P_hsp60_TK and *M. tuberculosis* wild-type strains respectively. Before imaging, 1 mCi of [^125^I]FIAU was injected into each mouse via a tail vein injection. Figure 3 shows [^125^I]FIAU-SPECT/CT images from the lungs of C3Heb/FeJ mice infected with either *M. tuberculosis* P_hsp60_TK or wild-type strains 6 or 8-weeks after a low-dose aerosol infection. SPECT activity was visualized in the lungs of mice infected with the *M. tuberculosis* P_hsp60_TK strain at both time-points (panels A, C). Though extensive lung disease was present, no SPECT activity was detected in the lungs of mice infected with the *M. tuberculosis* wild-type strain (panel B). As shown in panels C and D, the SPECT signal from the mouse infected with the *M. tuberculosis* P_hsp60_TK strain localized to the TB granuloma. *Ex-vivo* radioactivity in the selected granuloma was 5318 cpm per milligram of tissue compared with 2364 in lung tissue outside of the granuloma and 856 in lung tissue from the animal infected with *M. tuberculosis* wild-type strain.

## Discussion

Our overall goal was to develop a method to non-invasively detect and localize *M. tuberculosis* in experimentally-infected animals. We focused on using radiopharmaceutical-based imaging (CT, PET and SPECT) since it is already extensively used in humans. Moreover, unlike bioluminescence imaging (BLI), detection by radiopharmaceutical-based imaging is not limited by the location or depth of the signal. This means that bacteria or tissue deep inside the body or near the surface will be visualized equally well. Further, unlike some forms of BLI which require genetically modified mouse strains, radiopharmaceutical-based imaging techniques do not have such limitations, permitting their use in animal models with maximal relevance to human physiology. We have also recently shown that BLI is fraught with another significant problem, namely, that D-luciferin, the substrate used for imaging with firefly luciferase, is also a substrate for a multidrug (MDR) resistance pump in host-cells. Accordingly, differential expression of this MDR pump in the host-tissues substantially influences D-luciferin dependent bioluminescence *in vivo*, confounding the BLI readout [5]. It should however be noted that BLI imaging is likely to be more sensitive in detecting small numbers of bacteria than radiopharmaceutical-based imaging.

Though other investigators have used viral *tk* expression systems for FIAU imaging, we utilized a bacterial *tk* gene to ensure the likelihood of efficient translation and activity in *M. tuberculosis*. Moreover, we engineered the *M. tuberculosis* TK reporter strain utilizing a plasmid that integrates chromosomally and delivers only a single copy of the gene allowing more consistent expression. This method would also theoretically produce a more stable strain, and in this study, the *M. tuberculosis* P_hsp60_TK strain was stable even after several weeks of *in vivo* growth without antibiotic selection pressure. Moreover, these *in vivo* passaged strains maintained their [^125^I]FIAU uptake phenotype (data not shown). High levels of *tk* expression by the *P_hsp60_* could possibly be toxic to the mycobacteria, though this was not observed in this study. *M. tuberculosis* P_hsp60_TK growth characteristics and antimicrobial susceptibilities were similar to the wild-type parent strain. Finally, FIAU at high concentrations (>32 µg/ml) is known to inhibit growth of bacteria that have endogenous TK [2]. Standard dose of [^125^I]FIAU per animal of 2–5 mCi is equivalent to 0.34–0.85 µg of FIAU per animal. Since we have shown that FIAU concentration of up to 8 µg/ml have no significant affect on the *in vitro* growth of *M. tuberculosis* P_hsp60_TK strain, we did not anticipate any significant effect of FIAU on the TK strain *in vivo*. In fact, bacterial CFUs recovered from the lungs of mice infected with *M. tuberculosis* P_hsp60_TK strain after [^125^I]FIAU injection and SPECT imaging were not significantly different from those that were not administered [^125^I]FIAU (data not shown).

It has been previously reported that [^125^I]FIAU-SPECT can detect as few as 2 million *Staphylococcus aureus* per gram of tissue [2]. Our data from both the thigh and aerosol models suggests that [^125^I]FIAU-SPECT can detect as few as 5–10 million *M. tuberculosis* P_hsp60_TK inside a TB granuloma. This is significantly lower than the bacillary burden (10^7^–10^9^) observed in a cavitary TB granuloma during active TB in humans [6]. Therefore, this method would be useful in monitoring similar TB lesions in other animal models of TB such as guinea pigs, rabbits and NHP. However, by utilizing other radio-iodine isotopes (I-123, I-124), we can expect far greater (5–50 fold) sensitivity. Unfortunately, these isotopes are significantly more expensive. Use of more active mycobacterial promoters such as P*_A37_* and P_blaF*_ [7], [8] driving TK expression or TK substrates more specific for bacterial TK, may also increase sensitivity. Due to limited blood supply at the center of granulomas, it is possible that this site may be inaccessible to imaging substrates. However, our data suggests that FIAU was able to diffuse well into the necrotic granulomas. Finally, it should be noted that TK activity requires ATP. Quiescent bacteria may not trap significant amounts of FIAU and therefore may not be visualized well.

Non-invasive imaging technologies can be used for serial monitoring of TB disease in real-time in live animals. Since the same animals would be assessed at the different time-points, imaging would utilize significantly fewer animals. In other studies, we have shown that [^18^F]FDG-PET activity correlates with bactericidal activity of anti-TB regimens. Since [^18^F]FDG is marker of metabolic activity and inflammation, changes in [^18^F]FDG-PET activity temporally lagged the changes in bacterial burden [9]. Moreover, since inflammation generally lags bacterial burden during TB treatment [10], novel imaging biomarkers that directly label bacteria (FIAU) are anticipated to provide better real-time information. Non-invasive assessment would also be very useful for relapse studies where the time-interval between development of relapse and cessation of TB treatment is often unknown and large numbers of mice must be sacrificed at multiple time-points in order to have enough statistical power to detect differences in this binary endpoint.

Since the BALB/c mouse model lack well-defined granulomas that characterize human disease, we wanted to study [^125^I]FIAU-SPECT imaging in a model that develops caseous TB granulomas. To simulate these lesions we utilized the C3HeB/FeJ mouse strain that develops caseous necrosis in response to *M. tuberculosis* infection. We demonstrated that [^125^I]FIAU-SPECT signal localized to TB granulomas and confirmed these finding using post-mortem *ex vivo* tissue analysis.

The study of mycobacterial pathogenesis can be powerfully augmented by real-time observational capabilities of novel imaging biomarkers. Recently, imaging of *M. marinum* infection in the transparent zebrafish embryo has provided new insights into the temporal kinetics between mycobacteria and the host granuloma [11], [12]. However, zebrafish are not mammals and may therefore have distinct immunological responses to mycobacteria. Moreover, studies with *M. tuberculosis* rather than *M. marinum* are likely to be more relevant to human TB pathogenesis. More recently, Egen *et al*, used the mouse to produce the first live images of mycobacterial infection in a mammalian host [13]. Using transgenic mice and 3D time-lapse microscopy, the authors demonstrate that blood-borne BCG is rapidly taken up by Kupffer cells in the liver. Though extremely novel, these findings have limitations; unlike *M. tuberculosis*, BCG is an attenuated strain and imaging mouse liver granulomas induced after high-dose intravenous BCG challenge may be less relevant to human pathogenesis in lungs. Finally, Lewinsohn *et al* have shown that computed tomography provides real-time data on TB disease in monkeys, and has a tight correlation with post-mortem histopathology [14].

In summary, these studies provide proof-of-concept for application of [^125^I]FIAU-SPECT imaging as a novel method for preclinical and real-time pathogenesis studies in animal models of TB. Since [^125^I]FIAU-SPECT is a non-invasive technology, it will allow monitoring disease in the same group of animals and significantly reduce the numbers required per study. This will be essential to cost-effectively conduct TB studies in the larger and more expensive animal species (guinea pigs, rabbits, NHP) and accelerate TB drug and vaccine development. Moreover, this method will also allow real-time TB pathogenesis studies with virulent *M. tuberculosis* species in mammalian hosts, both of which are likely to be most relevant to human TB pathogenesis.
